# New Glutamine-Containing Substrates for the Assay of Cysteine Peptidases From the C1 Papain Family

**DOI:** 10.3389/fmolb.2020.578758

**Published:** 2020-10-22

**Authors:** Irina Y. Filippova, Elena A. Dvoryakova, Nikolay I. Sokolenko, Tatiana R. Simonyan, Valeriia F. Tereshchenkova, Nikita I. Zhiganov, Yakov E. Dunaevsky, Mikhail A. Belozersky, Brenda Oppert, Elena N. Elpidina

**Affiliations:** ^1^Division of Natural Compounds, Department of Chemistry, Moscow State University, Moscow, Russia; ^2^Department of Plant Proteins, A.N. Belozersky Institute of Physico-Chemical Biology, Moscow State University, Moscow, Russia; ^3^Laboratory of Protein Chemistry, Institute of Genetics and Selection of Industrial Microorganisms, Moscow, Russia; ^4^Division of Entomology, Faculty of Biology, Moscow State University, Moscow, Russia; ^5^USDA Agricultural Research Service, Center for Grain and Animal Health Research, Manhattan, KS, United States

**Keywords:** cathepsin B, cathepsin L, multicomponent enzyme mixtures, selective peptide substrates, *Tenebrio molitor*, *Tribolium castaneum*

## Abstract

New substrates with glutamine in the P1-position are introduced for the assay of peptidases from the C1 papain family, with a general formula of Glp-Phe-Gln-X, where Glp is pyroglutamyl and X is pNA (*p*-nitroanilide) or AMC (4-amino-7-methylcoumaride). The substrates have a simple structure, and C1 cysteine peptidases of various origins cleave them with high efficiency. The main advantage of the substrates is their selectivity for cysteine peptidases of the C1 family. Peptidases of other clans, including serine trypsin-like peptidases, do not cleave glutamine-containing substrates. We demonstrate that using Glp-Phe-Gln-pNA in combination with a commercially available substrate, Z-Arg-Arg-pNA, provided differential determination of cathepsins L and B. In terms of specific activity and kinetic parameters, the proposed substrates offer improvement over the previously described alanine-containing prototypes. The efficiency and selectivity of the substrates was demonstrated by the example of chromatographic and electrophoretic analysis of a multi-enzyme digestive complex of stored product pests from the Tenebrionidae family.

## Introduction

Cysteine peptidases constitute one of the main clans of proteolytic enzymes and are characterized by the presence of a Cys residue in the active center. Based on the MEROPS classification, the most numerous cysteine peptidase family is the papain C1 family (Rawlings et al., 2018). In the post-genomic era, C1 peptidases have been found in a variety of organisms – from viruses and protozoa to humans. Although the ancestor of the family is the plant peptidase papain, most attention has been paid to mammalian and human cysteine peptidases, called cathepsins. Currently, 11 human cysteine cathepsins are known: cathepsins B, C, F, H, K, L, O, S, L2 (alternative name V), X, and W, and the genes encoding these enzymes were found in a bioinformatics analysis of the human genome (Rossi et al., 2004). The main function of cysteine cathepsins is the non-specific degradation of proteins in lysosomes, but they also participate in more specific processes, such as cell cycle regulation, processing of enzymes and hormones, presentation of antigens, and formation of an immune response (Turk et al., 2012). The widespread involvement of lysosomal cathepsins in the processes of cellular proteolysis has led to their detailed study, especially in connection with involvement in the development of various pathological conditions, from rheumatoid arthritis to metastasis and growth of cancerous tumors (Repnik et al., 2012; Rudzińska et al., 2019; Soond et al., 2019).

Cysteine cathepsins are not only lysosomal peptidases, as there is clear evidence of their localization in other cellular compartments, such as the nucleus, cytoplasm and plasma membranes (Wex et al., 2003; Hsing and Rudensky, 2005; Salminen-Mankonen et al., 2007). Of particular interest are the cysteine cathepsins found in the multi-enzyme digestive complex of some insects (Vinokurov et al., 2005, 2006a,b, 2009; Semashko et al., 2014), as well as carnivorous plants (Ravee et al., 2018). Being digestive, these cathepsins not only have a higher degradomic potential than ordinary lysosomal cathepsins because of expanded substrates, but due to the structure of their natural substrates, they also exhibit specificity that is not characteristic of most known lysosomal cathepsins. Studies in the tenebrionid stored product pests *Tenebrio molitor* (yellow mealworm) and *Tribolium castaneum* (red flour beetle) indicated that they contain an expanded suite of 29 and 25, respectively, C1 cysteine cathepsins, and approximately half were predicted to be digestive (Tribolium Genome Sequencing Consortium, 2008; Morris et al., 2009; Martynov et al., 2015; Perkin et al., 2016). These digestive cysteine cathepsins were able to efficiently hydrolyze proline and glutamine-rich gliadins (wheat prolamins), the main storage proteins of seeds and the primary food substrates of these tenebrionids (Goptar et al., 2012). Understanding the prolamin-cleaving activity of cysteine cathepsins provided the prerequisites for their use as part of enzyme preparations for the treatment of celiac disease – a severe autoimmune hereditary disease caused by resistant to hydrolysis by human digestive peptidases glutamine- and proline-rich prolamin peptides (Piper et al., 2004; Shan et al., 2005). To successfully develop effective drugs containing cysteine peptidases, selective peptide substrates were necessary. However, a serious problem limiting the effectiveness of such studies was the lack of selective peptide substrates for this group of peptidases.

In this work we describe the development of selective and effective substrates of various cysteine peptidases of the C1 family, which contain a Gln residue at position P1, and thus are resistant to hydrolysis by trypsin-like peptidases.

## Materials and Methods

### Basic Methods for the Characterization of Synthesized Compounds

Thin-layer chromatography (TLC) was performed in the following systems: chloroform – methanol – acetic acid (45:5:1) (1) chloroform – methanol – acetic acid – water (75:15:5:2) (2); *n*-butanol-water-acetic acid (4:1:1) (3). TLC spots were detected with ninhydrin reagent (for compounds with a free α-amino group), chlorine – tolidine reagent and with UV lamp.

High-performance liquid chromatography (HPLC) chromatography was carried out with an Milichrom model A-02 chromatograph (EkoNova, Russia) using a ProntoSil 120-5C18AQ (2.0 mm × 75 mm) column eluted with a linear gradient of 0–80 % MeCN in water for 35 min at a flow rate 1 ml/min. The eluent contained 0.1% CF_3_COOH (Fluka AG). The elution profile was monitored at 214, 280, or 350 nm.

The amino acid composition was determined after 24 or 48 h hydrolysis with 5.7 M HCl at 105°C using a Hitachi 835 amino acid analyzer.

Mass spectra were obtained by a Finnigan LCQ-IOnTrap (Thermo Electron, United States) instrument using an electrospray ionization method.

NMR spectra were obtained in DMSO-D6 by a “Bruker AC-300” (Germany) spectrometer at the frequency of 400 Mhz. Chemical shifts were in parts per million (δ, ppm) toward the inner standard of tetramethylsilane.

### Chemical Synthesis

#### Boc-Gln-pNA

Five mL pyridine (61.7 mmol) and 10.5 mL *N*, *N*-diisopropylethylamine (60 mmol) were added by cooling to −10^*o*^C and stirring into a solution of 15 g of Boc-Gln-OH (60.3 mmol) in a mixture of 150 mL of dichloromethane (DCM) and 150 mL of tetrahydrofurane (THF). Pivaloyl chloride (7.5 mL, 60 mmol) was dissolved in 25 mL DCM and was added dropwise to the solution within a 15 min timeframe. The reaction mixture was stirred for 15 min at 0^*o*^C, and 9.12 g (66 mmol) of *p*-nitroaniline was added to the solution, which was stirred overnight at room temperature. The following day, the mixture was evaporated under reduced pressure. The residue was dissolved in a mixture of 200 mL ethyl acetate and 200 mL of 5% H_2_SO_4_, and the ethyl acetate layer was separated and washed successively with 200 mL of 10% KHSO_4_ (×2), 150 mL of a 20% solution of Na_2_SO_4_, 200 mL of a 5% solution of NaHCO_3_, and 150 mL of brine, followed by drying with anhydrous Na_2_SO_4._ The ethyl acetate solution was filtered from Na_2_SO_4_ and was concentrated *in vacuo* to yield an oily residue. The oil was dissolved in 300 mL of ethyl acetate, and 150 g of silica gel (70–230 mesh) was added to the solution. The mixture was stirred for 5 min, and the solution was filtered from the silica. The silica was washed with 150 mL ethylacetate (×3) and the combined ethylacetate solution was evaporated and crystalized with diethyl ether. The crystalline product was filtered, washed with a mixture of diethyl ether and petroleum ether and dried *in vacuo*.

Yield: 9.31 g (43 %). *R*_f_ 0.31 (1); 0.71 (2). *M*_r_ (calc./discov.) 366.4/366.4.

#### TFA × H-Gln-pNA

TFA × H-Gln-pNA was obtained from *Boc-Gln-pNA* by the standard procedure of deprotecting of Boc-group with trifluoracetic acid (TFA) (Goodman et al., 2004).

Yield 9.25 g (96 %). *R*_f_ 0.42 (3). *M*_r_ (calc./discov.). 380.8/380.8

#### Boc-Phe-Gln-pNA

TFA × H-Gln-pNA (3.0 g, 7.9 mmol) was added at 0^*o*^C to a solution of 4.0 g (9.2 mmol) of Boc-Phe-OPfp and 2.4 g (11.7 mmol) of sodium pentafluorophenolate in 50 mL DMF (Reakhim, Russia), further purified by the method described in Gordon and Ford (1972). The reaction mixture was stirred overnight at room temperature and was evaporated under reduced pressure. The oily residue was dissolved in a mixture of 100 mL ethyl acetate, 5 mL isopropanol, and 100 mL water. The organic layer was separated and washed successively with 100 mL of a 5% solution of H_2_SO_4_ (×2), 100 mL of a 20% solution of Na_2_SO_4_, 100 mL of a 5% solution of NaHCO_3_ (×2), and 100 mL of a saturated solution of NaCl, and was dried with anhydrous Na_2_SO_4_. The ethyl acetate solution was filtered from Na_2_SO_4_ and concentrated *in vacuo* to yield an oily residue. The oil was dried *in vacuo* to solid foam.

Yield: 3.6 g (90 %). *R*_f_ 0.36 (1); 0.80 (2).

#### TFA × H-Phe-Gln-pNA

TFA × Phe-Gln-pNA was obtained from *Boc-Phe-Gln-pNA* by standard procedure of deprotecting of Boc-group with TFA (Goodman et al., 2004).

Yield: 3.60 g (97 %). *R*_f_ 0.46 (3). *M*_r_ (calc./discov.) 527.4/527.4. ^1^H NMR, δ ppm: 10.86 (s, 1H); 8.95 (*d*, *J* = 7.3 Hz, 1H); 8.30 – 8.17 (m, 5H); 7.88 (*d*, *J* = 9.2 Hz, 2H); 7.36 (s, 1H); 7.28 – 7.15 (m, 5H); 6.85 (s, 1H); 4.49 (*q*, *J* = 6.9 Hz, 1H); 4.16 – 4.05 (m, 1H); 3.12 (dd, *J* = 9.1, 5.7 Hz, 1H); 2.96 (dd, *J* = 10.5, 7.6 Hz, 1H); 2.25 – 2.10 (m, 2H); 2.04 – 1.86 (m, 2H).

#### Glp-Phe-Gln-pNA

TFA (3.4 g, 6.5 mmol) × Phe-Gln-pNA was added to a solution of 2.1 g Glp-OPfp (7.3 mmol) (Lysogorskaya et al., 1983) and 2.1 g sodium pentafluorophenolate (10 mmol) in 50 mL of DMF at 0^*o*^C. The reaction mixture was stirred overnight at room temperature and evaporated under reduced pressure. The oily residue was dissolved in a mixture of 50 mL ethyl acetate, 50 mL 2-methyltetrahydrofurane, 5 mL iso-propanol, and 100 mL water. The organic layer was washed with 10% KHSO_4_, 20% Na_2_SO_4_, 5% NaHCO_3_ and a saturated solution of NaCl. The organic solution was stirred over anhydrous Na_2_SO_4_ for 1–2 min, filtered from Na_2_SO_4_ and concentrated *in vacuo* to yield an oily residue and dried *in vacuo* to a solid foam. The solid product was treated with ethyl acetate and the mixture was left overnight at 4^*o*^C. The crystalline product was filtered, the crystals were washed with ethylacetate and petroleum ether and dried *in vacuo*.

Yield: 1.76 g (52%).

#### Glp-Phe-Gln-AMC

The chemicals 0.059 g (0.21 mmol) Glp-Phe-OH (Lysogorskaya et al., 1983) and 0.081 g (0.21 mmol) HBr × Gln-AMC (Bachem, Switzerland) were dissolved in a mixture of 1.5 ml MeCN and 5 ml DMF, containing 30 μl (0.21 mmol) TEA (Reakhim, Russia), which was purified by the method described in Gordon and Ford (1972). To this solution, 0.054 g (0.26 mmol) DCC (Sigma-Aldrich) was added while cooling to 0°C and stirring. The reaction mixture was left at room temperature overnight, the precipitate that formed was filtered, and the filtrate was evaporated. The remaining oily substance was dissolved in a mixture of ethyl acetate – buthanol (80:20) (v/v). The organic layer was washed successively with 3% NaHCO_3_, water, 5% citric acid, water, and was dried over Na_2_SO_4_ and evaporated. The product crystallized after treatment with diethyl ether. The yield was 0,060 g (52%).

The physico-chemical characteristics of substrates Glp-Phe-Gln-pNA and Glp-Phe-Gln-AMC are presented in Supplementary Table 1.

#### Enzyme Assays of Cysteine Peptidases and Peptidases of Other Classes

The enzymatic activity of cysteine peptidases and peptidases of other classes was assayed with chromogenic and fluorogenic substrates. Papain (EC 3.4.22.2), ficin (EC 3.4.22.3), bovine cathepsin B (EC 3.4.22.1), human cathepsin L (EC 3.4.22.15), bovine trypsin (EC 3.4.21.4), and subtilisin Carlsberg (EC 3.4.21.62) were from Sigma-Aldrich (United States). Thermolysin (EC 3.4.24.27) and α-chymotrypsin (EC 3.4.21.1) were from Fluka AG (Switzerland). Porcine pepsin (EC 3.4.23.1) was purified as described (Solovjeva et al., 1977). *T. castaneum* cathepsin L was isolated and purified similar to the technique described below and in (Elpidina et al., 2019).

The activity of all peptidases was determined under optimal conditions for cysteine peptidases, and activity of serine, metallo- and aspartyl peptidases was additionally determined under optimal conditions of each enzyme. The activity of cysteine peptidases (papain, ficin, cathepsins B and L) and all other peptidases was measured at pH 5.6 in 0.1 M universal acetate-phosphate-borate buffer, (UB) (Frugoni, 1957), containing 1 mM EDTA and 6 mM Cys. The activities of serine peptidases (trypsin, chymotrypsin, subtilisin) and metallopeptidase thermolysin were also measured at pH 8.0 in 0.1 M Tris–HCl buffer, as well as the activity of aspartyl peptidase pepsin was measured at pH 3.5 in 0.1 M acetate buffer. Enzyme activities were measured at *D*_280_ 0.05–0.2 OU. Concentrations of papain, ficin, cathepsins B and L were determined as described in Semashko et al. (2014), using the titration of the active centers of enzymes by E-64, an irreversible inhibitor of cysteine peptidases. Trypsin concentration was similarly evaluated by data on titration of active sites. The concentrations of the solutions of peptidases were as follows: papain – 1.8–6.8 nM; ficin – 4.9–24.8 nM; cathepsin B – 9.4–46 nM; *T. castaneum* cathepsin L – 23 – 67 nM; cathepsin L human – 16 nM; trypsin – 76 nM; chymotrypsin – 49 nM; subtilisin – 160 nM; thermolysin – 13 nM; pepsin – 47 nM.

#### Enzyme Assays Using Chromogenic (pNA) Substrates

For assays, 5 μl of enzyme solution were added to wells of a 96-well plate and diluted to a final volume of 195 μl with suitable buffer. The mixture was preincubated at room temperature for 15 min and 5 min at 37°C before the addition of substrate. To initiate the reaction, 5 μl of 20 mM substrate stock solution in DMF was added and initial absorption at 405 nm at time zero was measured and thereafter every 3 min for 60 min at 37°C in a microplate reader (ELx808, BioTek, United States).

Enzymatic activity was calculated by the formula:

$$A=k_{\text{chr}}\,\frac{d\,D_{405}}{d\,t}\,\frac{1}{c_{\text{enz}}}\times 10^{-6}$$

where *A* is the specific activity of the preparation, *M*_substrate_ × *M*^–1^_enzyme_ × min^–1^; *dD*_405_/*dt*, OU × min^–1^, is an initial rate of *p*-nitroaniline release, determined as a coefficient of initial linear plot of kinetic curve (a function of the change in absorbance at 405 nm over time); *k*_chr_ = 157 μM × OU^–1^ – concentration of *p*-nitroaniline at which the absorbance of the solution is equal to 1 optical unit (OU) (defined in a separate experiment by plotting a calibration curve); *c*_enz_ – enzyme concentration in the sample, M.

#### Enzyme Assays Using Fluorogenic (AMC) Substrates

The enzymatic activity with fluorogenic substrates was assayed similar to that described for pNA substrates, using 1 mM substrate stock solutions in DMF. The fluorescent measurements were performed in a 96-well microplate and were measured in a spectrofluorimeter (FL × 808, BioTek, United States) at λ_ex_ = 355 nm and λ_em_ = 440 nm.

Enzymatic activity was calculated by the formula:

$$A=k_{\text{fl}}\,\frac{d\,I}{d\,t}\,\frac{1}{C_{\text{enz}}}\times 10^{-6}$$

where *A* is the specific activity of the preparation, *M*_substrate_ × M^–1^_enzyme_ × min^–1^; *dI*/*dt*, U × min^–1^, is an initial rate of fluorophore release, determined as a coefficient of a linear plot of the kinetic curve (a function of fluorescence intensity I from time); *k*_fl_, μM × U^–1^. – concentration of a fluorophore at which the fluorescence intensity of the solution is equal to 1 unit (U) (defined in a special experiment by plotting a calibration curve), *k*_fl_ = 0.02 μM × U^–1^; *c*_enz_ – enzyme concentration in the sample, M.

### Determination of Kinetic Parameters of Substrate Hydrolysis

The initial rates of hydrolysis of the chromogenic substrates were determined according to (Semashko et al., 2014) by similar procedure as described above with substrate concentrations of 0.05 – 0.5 mM. The ratio *k*_cat_/*K*_M_ was calculated from the values of kinetic parameters obtained by nonlinear regression using the program OriginPro 7.5 or by linearization of the standard Michaelis-Menten equation by Hanes-Woolf and Lineweaver-Burk plots (Dixon and Webb, 1982) using Microsoft Excel. Kinetic measurements were performed 3–5 times in triplicate. Calculations of *k*_cat_/*K*_M_ were carried out by least squares regression using Microsoft Excel 2016.

### Native PAGE and Post-electrophoretic Detection of Proteolytic Activity

The extract of *T. molitor* larvae midguts was obtained as previously described (Vinokurov et al., 2006a,b). Briefly, fourth instar *T. molitor* larvae that were actively feeding on high-temperature processed milled oat flakes were immobilized on ice, and the gut was removed in 0.15 M NaCl. The gut was washed in precooled distilled water and was divided into two equal parts, anterior midgut (AM) and posterior midgut (PM), and each was homogenized in MilliQ (Millipore, Molsheim, France) water in a glass Downce homogenizer (50 AM or PM parts in 350 μl of water). The homogenate was centrifuged for 5 min at 10,000 × *g*. The resulting supernatant was used further for electrophoretic studies or stored for 1–2 days at −70°C until use.

Native PAGE of crude extract of *T. molitor* larvae midguts and post-electrophoretic detection of proteolytic activity was performed as described in Elpidina et al. (2019). Extract in the amount of 0.2 larval gut equivalent (2 μl of extract) was applied to each electrophoretic well. The concentration of E-64 was 10^–5^ M in samples with an inhibitor. The electrophoresis was performed toward the anode at 10 mA constant current and 4°C in 1-mm thick 12% polyacrylamide gels (Bio-Rad Mini Protean 3 system) with 35 mM HEPES and 43 mM imidazole buffer, pH 7.2, according to (McLellan, 1982). Proteolytic activity was detected with 125 μM fluorogenic substrates Glp-Phe-Gln-AMC, Z-Phe-Arg-AMC (Bachem, Switzerland), Z-Arg-Arg-AMC (Bachem, Switzerland) at pH 5.6 (0.1 M Universal acetate–phosphate–borate buffer, UB, Frugoni, 1957), in the presence of 6 mM cysteine and 1 mM EDTA (Sigma-Aldrich) or at pH 7.9 (0.1 M UB).

### Fractionation of Digestive Peptidases From *T. castaneum* Larvae

From stock cultures of *T. castaneum*, larvae were dissected as described in Vinokurov et al. (2009). Entire guts were excised, pooled and homogenized in 0.15 M NaCl in a glass Downce homogenizer, with 2.1 ml of extract (*D*_280_ = 5.9) obtained from 245 guts of *T. castaneum* larvae. Pooled extracts were centrifuged at 16,000 rpm for 1 h at 4°C and incubated for 30 min with 10^–4^ M TLCK (*N*-α-tosyl-L-lysine-chloromethyl ketone) (Bachem, Switzerland) and 10^–4^ M chymostatin (Sigma-Aldrich, United States). The clarified extract was applied to a column with Sephadex G-75 (Pharmacia, Sweden) (2.5 × 120 cm) in 0.01 M phosphate buffer, pH 5.6 at 4°C. Fractions of 2.2 ml were collected. The protein concentration in the fractions was estimated by the optical density at 280 nm. Enzymatic activity in the fractions of the eluate was determined in 0.1–0.18 ml aliquots using the substrates Glp-Phe-Gln-pNA, Z-Phe-Arg-pNA, Z-Arg-Arg-pNA at pH 5.6 in the presence of 6 mM cysteine and 1 mM EDTA according to the microplate assay described above.

## Results

### Design of Substrates

The structure of the newly synthesized substrates can be expressed by the general formula Glp-Phe-Gln↓X where Glp is pyroglutamyl; *X* = pNA (*p*-nitroanilide) or AMC (4-amino-7-methylcoumaride); the location of the hydrolysis site is indicated by the arrow. The structure of the substrates meets the requirements of the specificity of cysteine peptidases from the C1 family: a key P1 position occupies a medium sized Gln residue, and in position P2, which is decisive for the specificity of these peptidases, is a hydrophobic Phe residue (Harris et al., 2000; Choe et al., 2006). The N-terminal group of substrates is represented by the residue Glp, providing better solubility in aqueous-organic mixtures. As C-terminal residues (X) are chromogenic *p*-nitroanilide and fluorogenic 4-amino-7-methylcoumaride groups, they ensure easy and precise control of enzyme activity during substrate hydrolysis.

### Synthesis of Substrates

The synthesis of the substrates was performed by fragment condensation using carbodiimide as a condensing agent or an activated ester method using pentafluorophenol or sodium pentafluorophenolate. The latter is especially useful in the preparation of Gln-containing peptides, the synthesis of which is complicated by adverse reactions, in particular, rearrangement of Gln residue into pyroglutamic acid residue. The contribution of adverse reactions is most significant under the conditions of formation of the free bases of Gln derivatives (Mazurov et al., 1997).

To obtain pyroglutamyl derivatives we used an approach based on the use of GlpOP_fp_ (pyroglutamyl pentafluorphenyl ether) (Semashko et al., 2008). Peptide condensation was carried out in anhydrous organic solvents DMF (*N*, *N*-dimethylformamide) and MeCN (acetonitrile), adding into the reaction equimolar amounts of the carboxyl and amine components, and 5–10% excess of DCC (*N*, *N*′-dicyclohexylcarbodiimide).

The newly synthesized substrates were characterized by mass-spectrometry, chromatographic properties [mobility values in several systems (TLC) and retention times (HPLC)], NMR data, and amino acid analysis (Supplementary Table 1).

The resulting compounds Glp-Phe-Gln-pNA and Glp-Phe-Gln-AMC were compared to alanine-containing selective substrates, Glp-Phe-Ala-pNA and Glp-Phe-Ala-AMC, that were synthesized and described in our laboratory earlier (Semashko et al., 2014), in terms of their hydrolysis efficiency by the studied cysteine peptidases. Two parameters were compared: (1) specific activity (*M*_substrate_/*M*_enzyme_^∗^ min) (Table 1) and (2) specificity constant *k*_cat_/*K*_M_ (Table 2) which characterizes both the rate of the enzymatic reaction (*k*_cat_ value) and the affinity of a given enzyme to a given substrate (*K*_M_ value).

**TABLE 1:** Specific activity (*M*_substrate_/*M*_enzyme_*min) of cysteine peptidases from plants and animals with the chromogenic substrates Glp-Phe-Ala-pNA and Glp-Phe-Gln-pNA, and the fluorogenic substrates Glp-Phe-Ala-AMC and Glp-Phe-Gln-AMC (*n* = 3, ±SE).

**Enzyme**	**Chromogenic substrates**		**Fluorogenic substrates**	
	**Glp-Phe-Ala-pNA**	**Glp-Phe-Gln-pNA**	**Glp-Phe-Ala-AMC**	**Glp-Phe-Gln-AMC**
Papain	190 ± 1	410 ± 4	250 ± 1	620 ± 15
Ficin	68 ± 1	96 ± 2	63 ± 1	140 ± 10
Cathepsin B bovine	15 ± 1	21 ± 2	7 ± 1	23 ± 9
Cathepsin L human	22 ± 1	68 ± 1	18 ± 1	110 ± 1
Cathepsin L *Tribolium castaneum*	27 ± 1	69 ± 1	15 ± 1	97 ± 9
**Serine, metallo- and aspartic peptidases**				
Chymotrypsin	0	0	0	0
Trypsin	0	0	0	0
Subtilisin	0.100 ± 0.001 (0.510 ± 0.002)*	0.050 ± 0.001 (0)*	0	0
Thermolysin	0	0	0	0
Pepsin	0	0	0	0

*The values of specific activity in parentheses were measured under conditions optimal for subtilisin (see section “Enzyme Assays of Cysteine Peptidases and Peptidases of Other Classes”).

**TABLE 2:** Efficiency of the hydrolysis of substrates Glp-Phe-Ala-pNA and Glp-Phe-Gln-pNA by cysteine peptidases of C1 family (*n* = 3, ±SE).

**Enzyme**	**Substrate**	***k*_cat_/*K*_M_, mM^–1^ min^–1^**
Papain	Glp-Phe-Gln-pNA	1,410 ± 150
	Glp-Phe-Ala-pNA	640 ± 80
Ficin	Glp-Phe-Gln-pNA	490 ± 50
	Glp-Phe-Ala-pNA	180 ± 20
Cathepsin B human*	Glp-Phe-Gln-pNA	44 ± 2
	Glp-Phe-Ala-pNA	34 ± 2
Cathepsin L *T. castaneum*	Glp-Phe-Gln-pNA	190 ± 24
	Glp-Phe-Ala-pNA	33 ± 4
Cathepsin L human*	Glp-Phe-Gln-pNA	140 ± 27
Cathepsin L human**	Glp-Phe-Ala-pNA	33 ± 4

*Recombinant preparation. **From Semashko et al. (2014).

### Hydrolysis of Substrates

Hydrolysis of the new glutamine-containing substrates was compared to that of the previous alanine-containing substrates (Stepanov et al., 1985; Semashko et al., 2014) using C1 family cysteine peptidases: the plant enzymes papain, bromelain, and ficin, lysosomal mammalian cathepsins B and L, as well as digestive cathepsin L from *T. castaneum* larvae (Coleoptera: Tenebrionidae). The substrates were cleaved by all tested cysteine peptidases (Table 1). In general, the specific activity with Gln-containing substrates was higher for all peptidases as compared to that of Ala-containing analogs. All substrates were most efficiently cleaved by plant cysteine peptidases. Among the cathepsins of animal origin, cathepsin L from both human and insect had higher specific activities with all substrates.

Catalytic efficiency of enzymatic reaction with the substrates Glp-Phe-Gln-pNA and Glp-Phe-Ala-pNA was estimated by the specificity constant of enzymatic reaction *k*_ca__t_/*K*_M_ (Table 2). All peptidases showed higher specificity constants for the substrate Glp-Phe-Gln-pNA compared to Glp-Phe-Ala-pNA. The highest constants were demonstrated by plant peptidases, especially by papain. However, the superiority of the Gln-containing substrate compared to its Ala-containing analog was most noticeable in the case of cathepsin L (about 4-6-fold).

### Substrates Selectivity

The newly synthesized glutamine containing substrates were compared according to their class selectivity (Table 1). We tested the hydrolysis of both the chromogenic and fluorogenic substrates by commercial preparations of peptidases from different clans (classes): serine peptidases: trypsin, α-chymotrypsin, subtilisin Carlsberg; aspartyl peptidase pepsin; and metallopeptidase thermolysin. Enzyme activity was measured under conditions optimal for cysteine peptidases: in weakly acidic medium in the presence of the reducing agent cysteine, as well as under conditions optimal for each of the enzymes. The concentrations of all peptidases were comparable, except for subtilisin, which was several times higher than the concentrations of cysteine peptidases. Very low activity of subtilisin for Glp-Phe-Ala-pNA and practically zero activity against Glp-Phe-Gln-pNA was observed. Thus, the data indicate that all substrates were selective for cysteine peptidases from the C1 family and were not hydrolyzed by peptidases of other clans. This selectivity provided an important advantage with respect to common commercially available arginine-containing substrates: Z-Phe-Arg-pNA, Z-Phe-Arg-AMC, Z-Arg-Arg-pNA, and Z-Arg-Arg-AMC, that are actively cleaved also by trypsin-like serine peptidases (Semashko et al., 2014). With this selectivity, the glutamine-containing substrates can be used to evaluate cysteine peptidase activity in the mixtures containing enzymes from other clans.

We illustrated the selectivity of the new substrates for cysteine peptidases using a natural multi-component enzyme system - digestive peptidases of stored product pests from the Tenebrionidae family, *T. castaneum* and *T. molitor*, containing a diverse mixture of cysteine and serine peptidases (Vinokurov et al., 2006a,b, 2009; Morris et al., 2009; Martynov et al., 2015; Perkin et al., 2016). The digestive organ in these insects is the midgut and is demarcated by two physiologically different sections, the anterior midgut (AM) with a pH of 5.6 in both insects, and the posterior midgut (PM) with an average pH of 7.5 in *T. castaneum* and 7.9 in *T. molitor* (Vinokurov et al., 2006a,b, 2009). This research has demonstrated that the acidic anterior midgut contains mainly cysteine peptidases, whereas serine peptidases are more represented in the posterior midgut of both insects.

**FIGURE 1:**
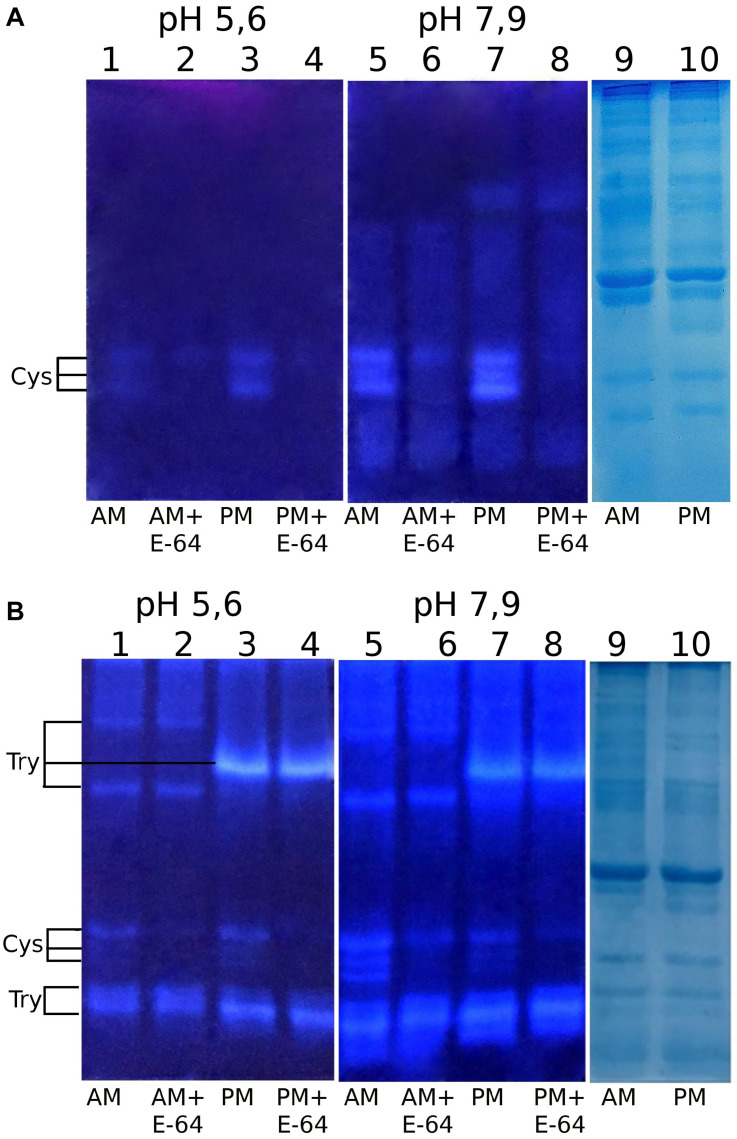
Post-electrophoretic visualization of the activity of peptidases from the midgut extracts of *T. molitor* larvae after native PAGE using the newly synthesized substrate Glp-Phe-Gln-AMC **(A)** and a commercial substrate Z-Phe-Arg-AMC **(B)**: 1 – anterior midgut, pH 5.6; 2 – anterior midgut + E-64, pH 5.6; 3 – posterior midgut, pH 5.6; 4 – posterior midgut + E-64, pH 5.6; 5 – anterior midgut, pH 7.9; 6 – anterior midgut + E-64, pH 7.9; 7 – posterior midgut, pH 7.9; 8 – posterior midgut + E-64, pH 7.9; 9,10 – Coomassie G-250 staining, anterior and posterior midgut, respectively. Cys – activity of cysteine peptidases; Try – activity of trypsin-like peptidases.

Using our method of post-electrophoretic detection of peptidase activity (Elpidina et al., 2019), we were able quickly and easily separate and selectively identify cysteine peptidases in the crude midgut extract of *T. molitor* (Figure 1). Electrophoretic separation of the midgut extract containing digestive peptidases of *T. molitor* larvae was under native conditions at either pH 5.6 or 7.9. Activity tested with the commercial substrate Z-Phe-Arg-AMC was non-selective and resulted in the visualization of both serine trypsin-like and cysteine peptidases. Therefore, these enzyme activities were distinguished only by using an appropriate concentration of the cysteine peptidase inhibitor E-64, requiring additional experimental steps. The newly synthesized fluorogenic substrate Glp-Phe-Gln-AMC was selective and detected the activity of cysteine peptidases exclusively. In addition, Glp-Phe-Gln-AMC, like Z-Phe-Arg-AMC, was highly efficient. Therefore, Glp-Phe-Gln-AMC was able to identify cysteine peptidases in lower concentrations that cannot be determined by staining with Coomassie G-250. Thus, the use of a combination of substrates made it possible to separately detect serine and cysteine peptidases without using specific inhibitors.

**FIGURE 2:**
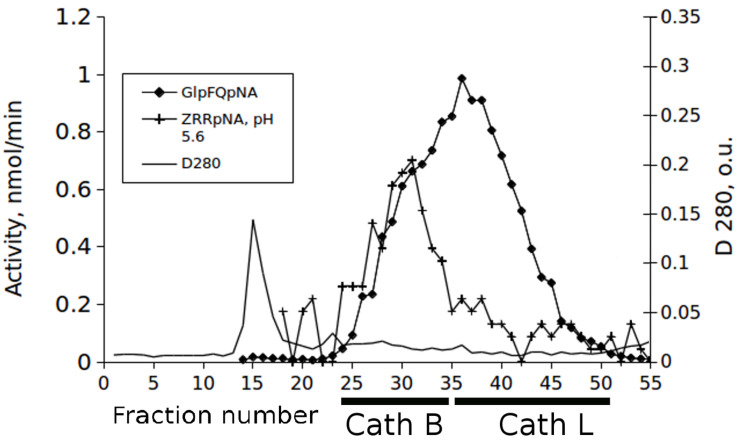
The elution profiles of cysteine peptidase activities in the extract of the *T. castaneum* larvae guts after fractionation on the Sephadex G-75 column with the substrates Glp-Phe-Gln-pNA (GlpFQpNA) and Z-Arg-Arg-pNA (ZRRpNA) at pH 5.6 and 6 mM cysteine added to the buffer.

Identification of the activity of cathepsins L and B in chromatographic separations of midgut extracts from *T. castaneum* larvae was made with the newly synthesized chromogenic substrate Glp-Phe-Gln-pNA in combination with the commercial substrate Z-Arg-Arg-pNA. The substrate Glp-Phe-Gln-pNA is a common substrate for all cysteine peptidases of the C1 family but was cleaved at higher efficiency by cathepsin L than by cathepsin B (Tables 1, 2). The substrate Z-Arg-Arg-pNA is hydrolyzed by cathepsin B and is cleaved at much lower efficiency by cathepsin L (Semashko et al., 2014). Thus, using Glp-Phe-Gln-pNA and Z-Arg-Arg-pNA simultaneously permitted the differentiation of cysteine cathepsins L and B (Figure 2).

## Discussion

A wide variety of modified peptides – peptidomimetics – have been described as substrates for evaluating the enzymatic activity of cysteine peptidases of the C1 family, of which the distinguishing feature is the presence of Arg residue at P1 position (Lowe and Yuthavong, 1971; Mole and Horton, 1973; Brubacher and Zaher, 1979; Zucker et al., 1985; Tchoupe et al., 1991; Ménard et al., 1993; Melo et al., 2001; Cezari et al., 2002; Puzer et al., 2004; Turk et al., 2012). However, the Arg residue is also recognized by trypsin-like peptidases, and therefore substrates with Arg are not selective for the recognition of cysteine peptidases.

Another approach is based on the synthesis of peptide libraries for identifying cysteine cathepsin activity (Cotrin et al., 2004; Choe et al., 2006; Ruzza et al., 2006). This approach allowed rapid screening of enzymatic activity, but due to the ambiguity of the results, has not replaced standard enzyme studies using multiple sets of substrates. Currently, only a very limited number of studies have been devoted to the development of synthetic substrates for certain types of cysteine peptidases. Therefore, substrates were developed for cathepsin B (Cezari et al., 2002; Ruzza et al., 2006), cathepsin L (Puzer et al., 2004; Lęgowska et al., 2014), cathepsin C (Lęgowska et al., 2016), and cathepsin V (Puzer et al., 2004) activities, but all are also non-selective.

Thus, the main drawback of the available synthetic peptide substrates of cysteine peptidases is their non-selectivity. This limitation significantly diminishes the sensitivity and correct determination of the activity of this group of enzymes. We previously proposed a selective chromogenic substrate Glp-Phe-Leu-pNA, but this compound had low solubility in water (Filippova et al., 1984). We further synthesized and characterized a selective chromogenic substrate Glp-Phe-Ala-pNA with improved solubility and its fluorogenic analogs, Glp-Phe-Ala-AMC (AMC – 4-amino-7-methylcoumaride) and Glp-Phe-Ala-AFC (AFC – 4-amino-7-fluoromethyl-coumaride) (Stepanov et al., 1985; Semashko et al., 2008, 2014). These alanine-containing peptidomimetics were selective for cysteine peptidases, and could be used for testing activity during the purification and characterization of cysteine peptidases in complex multi-component natural mixtures like extracts of insect digestive peptidases (Vinokurov et al., 2005, 2006a,b, 2009) and for direct detection of unstable cysteine peptidase activity in polyacrylamide gels after native electrophoresis (Elpidina et al., 2019). However, these substrates had a lower rate of hydrolysis than commercially available arginine-containing substrates Z-Phe-Arg-pNA, Z-Phe-Arg-AMC, and Z-Arg-Arg-pNA, Z-Arg-Arg-AMC.

The results presented in this paper demonstrate that Gln-containing substrates have improved kinetic characteristics over our previous Ala-containing substrates. The most significant advantage of glutamine-containing substrates compared to the Ala-containing prototypes is their selectivity not only for peptidases of other clans, but also for various cysteine peptidases inside one group. Chromatographic separation of cysteine peptidases from an extract of the *T. castaneum* larvae gut demonstrate that by using the glutamine-containing chromogenic selective peptide substrate Glp-Phe-Gln-pNA in combination with commercially available Z-Arg-Arg-pNA, it was possible to reliably differentiate two different cysteine cathepsins - cathepsin L and cathepsin B. Z-Arg-Arg-pNA is a known substrate of cathepsin B, and cathepsin L enzymes are more specific than cathepsin B for Glp-Phe-Gln-pNA. Cathepsins B and L eluted in different fractions during our gel-filtration chromatography, as was visualized by means of activity testing with this pair of substrates. The prototype peptide substrate of the papain family peptidases, Glp-Phe-Ala-pNA, was only slightly more specific for cathepsin B than cathepsin L (Semashko et al., 2014), so only the use of a glutamine-containing substrate was able to differentiate major cathepsins B and L in one cycle.

Because cysteine cathepsins are involved in human and other animal diseases, as well as critical to many life processes, the development of selective and efficient substrates for their study is a major improvement in the study of these important enzymes.

## Conclusion

The newly produced selective substrates for cysteine peptidases containing Gln in the P1 position have better kinetic characteristics than the previously described Ala-containing selective substrates. The major advantage of the Gln-containing substrates is the selectivity for cysteine peptidases compared to non-selective commercially available substrates, containing Arg in P1 position. The new substrates allow the differentiation of the activity of cysteine peptidases in complex multi-component natural mixtures containing peptidases from different classes, as well as the distinction of cysteine cathepsins L and B.

## Data Availability Statement

All datasets presented in this study are included in the article/Supplementary Material.

## Author Contributions

IF designed and coordinated the study, designed substrates, analyzed data, and wrote the manuscript. ED performed experiments to study the hydrolysis of substrates, performed chromatographic separation of the extract of *T. castaneum* larvae, and wrote the manuscript. NS synthesized and characterized substrates. TS and VT performed experiments to study the hydrolysis of substrates and obtained kinetic parameters of hydrolysis. NZ performed PAGE. YD, MB, and BO edited the manuscript. EE coordinated the study, analyzed the results, and wrote the manuscript. All authors contributed to the article and approved the submitted version.

## Conflict of Interest

The authors declare that the research was conducted in the absence of any commercial or financial relationships that could be construed as a potential conflict of interest.
