# Supplementary material for: New Glutamine-Containing Substrates for the Assay of Cysteine Peptidases From the C1 Papain Family
Source: Front Mol Biosci. 2020 Oct 22;7:578758. doi: 10.3389/fmolb.2020.578758 (PMC7643032; doi:10.3389/fmolb.2020.578758)
Supplement: Supplementary file 1 [file Table_1.DOCX]

Supplementary Material

# Supplementary Data

**Supplementary Table 1.** Physico-chemical characteristics of synthesized substrates.

| Substrate | Amino Acid Analysis^1^ | TLC^2^_,_  R*_f_* | HPLC^7^_,_  t_R_,min | M_r_^8^ (calc./discov.) | NMR^1^H, DMSO-D_6_, δ, ppm^9^ |
| --- | --- | --- | --- | --- | --- |
| Glp-Phe-Gln-pNA | Glu:Phe 1.98:1 | 0.15^3^  0.47^4^ | 16.7 | 524.5/524.5 | 1.93 – 2.06 (m, 4H, Gln-βСН_2_, Glp-βСН_2_); 2.13 – 2.25 (m, 4H Gln-γСН_2_, Glp-γСН_2_); 2.83 (dd, *J* = 13.5, 10.4 Hz, 1H, Phe-СН_2_); 3.13 (dd, *J* = 9.9, 3.5 Hz, 1H, Phe-СН_2_); 3.98 (dd, *J* = 8.5, 5.1 Hz, 1H, Glp-αСН); 4.39 (q, *J* = 7.1 Hz, 1H, Gln-αСН); 4.50 – 4.59 (m, 1H, Phe-αСН); 6.84 (d, *J* = 6.7 Hz, 1H, Phe-NH); 7.21 (t, *J* = 7.2 Hz, 2H, Gln-CONH_2_); 7.28 – 7.64 (m, 5H, Phe-C_6_H_5_); 7.96 (d, *J* = 9.2 Hz, 2H, pNA-CH); 8.22 (d, *J* = 9.2 Hz, 2H, pNA-CH); 8.38 (d, *J* = 8.6 Hz, 1H, Glp-NH); 9.09 (d, *J* = 6.7 Hz, 1H, Gln-NH); 11.32 (s, 1H, pNA-NH). |
| Glp-Phe-Gln-AMC | Glu:Phe 2.1:1 | 0.77^5^  0.85^6^ | 13.5 | 561.5/561.4 | 1.81 (s, 3H, AMC-СН_3_); 1.90 – 2.04 (m, 4H, Gln-βСН_2_, Glp-βСН_2_); 2.11 – 2.24 (m, 4H Gln-γСН_2_, Glp-γСН_2_); 2.85 (dd, *J* = 13.2, 10.2 Hz, 1H, Phe-СН_2_); 3.14 (dd, *J* = 10.1, 3.6 Hz, 1H, Phe-СН_2_); 4.01 (dd, *J* = 8.4, 5.0 Hz, 1H, Glp-αСН); 4.42 (q, *J* = 7.1 Hz, 1H, Gln-αСН); 4.52 – 4.62 (m, 1H, Phe-αСН); 6.18 (s, 1H, AMC-СНCO); 6.91 (d, *J* = 6.8 Hz, 1H, Phe-NH); 7.15 (d, *J* = 9.1 Hz, 1H, AMC-CH); 7.24 (t, *J* = 7.3 Hz, 2H, Gln-CONH_2_); 7.30 – 7.65 (m, 5H, Phe-C_6_H_5_); 7.71 (s, 1H, AMC-СН); 8.09 (d, *J* = 9.1 Hz, 1H, AMC-CH); 8.27 (d, *J* = 8.7 Hz, 1H, Glp-NH); 8.98 (d, *J* = 6.6 Hz, 1H, Gln-NH); 10.45 (s, 1 H, AMC-NH). |

^1^The molar ratio of amino acid residues. Amino acid analyses were performed on a Hitachi 835 (Japan) automatic amino acid analyzer after acidic hydrolysis of samples with 5.7 M HCl at 105°C in evacuated ampoules for 24 and 48 h. ^2^TLC spots were detected with UV lamp and chlorine – tolidine reagent. ^3^In chloroform – methanol – acetic acid (45:5:1). ^4^In chloroform – methanol – acetic acid – water (75:15:5:2).^5^In *n*-butanol-water-acetic acid (4:1:1). ^6^In *n*-butanol-pyridine-water-acetic acid (15:12:10:3). ^7^HPLC chromatography was carried out with an Milichrom model A-02 chromatograph (EkoNova Russia) using a ProntoSil 120-5C18AQ (2.0 × 75 mm) column eluted with a linear gradient of 0-80 % MeCN (for HPLC, Lekbiofarm (Russia)) in water for 35 min at a flow rate 1 ml/min. The eluent contained 0.1% TFA (Fluka AG). The elution profile was monitored at 214, 280 or 350 nm. ^8^Mass spectra were obtained by a Finnigan LCQ-IOnTrap (Thermo Electron, USA) instrument using an electrospray ionization method. ^9^NMR spectra were obtained in DMSO-D6 by a "Bruker АС-300" (Germany) spectrometer at the frequency of 400 Mhz. Chemical shifts were in parts per million (δ, ppm) towards the inner standard of tetramethylsilane.
